# Screening of high *β*-glucosidase-producing yeast strains from the Penglai wine region (China) and their fermentation performances and aroma compositions in Petit Manseng wine fermentation

**DOI:** 10.3389/fmicb.2025.1653569

**Published:** 2025-08-11

**Authors:** Xiao Hong Tang, Yan Ding, Ke Zhong, Yu Xia Sun, Xiao Mei Han, Zhi Yu Li, Rui Rui Li

**Affiliations:** Shandong Academy of Grape, Jinan, Shandong, China

**Keywords:** *β*-glucosidase, non-*Saccharomyces cerevisiae*, Terpenes, C13-norisoprenoids, *H. occidentalis*, *M. andauensis*

## Abstract

The majority of aroma precursors in grapes exist as odorless glycosidic conjugates, which can be hydrolyzed by *β*-glucosidase to release free volatile aroma compounds, thereby enhancing the aromatic quality of wine. This study aimed to screen and characterize indigenous non-*Saccharomyces cerevisiae* strains with *β*-glucosidase activity for their potential to enhance terpenoid aroma compounds during wine fermentation. Grapes collected from 14 vineyard plots in the Penglai Wine Region (China) underwent spontaneous fermentation and yielded 203 single colonies. Among them, 85 strains of non-*Saccharomyces cerevisiae* were initially screened based on the geniposide chromogenic method, which were classified into 7 genera and 16 species. Nine high-performance strains were subsequently selected for small-scale fermentation trials using Petit Manseng grape juice. The physicochemical parameters of wines were analyzed by high-performance liquid chromatography (HPLC), while volatile aroma compounds were quantified using a headspace solid-phase microextraction coupled with gas chromatography–mass spectrometry (HS-SPME-GC–MS). The results revealed that *Starmerella bacillaris* CGCD1-9, *Pichia fermentans* CGCD1-4, and *Zygosaccharomyces bailii* JDCD01 significantly enhanced glycerol production, which might contribute to improved wine sensory quality. Four *Hanseniaspora* yeast strains exhibited the ability to increase ethyl acetate content. Among these strains, *Hanseniaspora vineae* CGCD1-1, *Hanseniaspora uvarum* CGCD1-3, and *Hanseniaspora opuntiae* CGCD1-7 showed a pronounced tendency to elevate geraniol levels, whereas *Hanseniaspora occidentalis* CGCD1-5 selectively promoted the biosynthesis of *β*-damascenone and 2-furanmethyl acetate. *Zygosaccharomyces bailii* JDCD01 primarily increased the levels of isobutanol and phenylethyl alcohol, while also exhibiting a slight enhancement in terpenoid production. Notably, *S. bacillaris* CGCD1-9 significantly enhanced five key terpenoids, namely, linalool, geraniol, citronellol, *α*-terpineol, and nerolidol, yielding the highest total terpene content among all strains, while increasing the content of alcohols (less than JDCD01). In contrast, *Issatchenkia terricola* SIVE4101 and *Metschnikowia andauensis* LFSY0-17 showed preferential accumulation of *β*-damascenone and total furans, with the former also significantly increasing the acetate ester content. This study provides new ideas and theoretical support for r developing targeted co-inoculation protocols with *S. cerevisiae* to achieve precise modulation of wine terpenoid profiles.

## 1 Introduction

Aroma represents a critical organoleptic parameter for assessing wine quality. Based on their origin, wine aromas can be systematically categorized into three distinct groups: (1) varietal aromas (primary aromas derived from grapes), (2) fermentation aromas (secondary aromas produced during alcoholic fermentation), and (3) aging aromas (tertiary aromas developed during maturation; Vararu et al., 2016; Belda et al., 2017).

The varietal aroma profile is predominantly composed of three major classes of volatile compounds: terpenes, C13-norisoprenoids, and volatile sulfur compounds. Among these classes, terpenoids are particularly noteworthy due to their low sensory thresholds and characteristic floral/fruity notes. These properties make terpenoids significant biomarkers for determining the typicity of both grapes and their corresponding wines (Maria et al., 2021). Most aromatic compounds exist in a glycosidically bound form, which is odorless and imperceptible (Sharp et al., 2017; Graf et al., 2022). To enhance and improve the aromatic profile of wine, it is essential to convert these bound aroma precursors into their free volatile forms. *β*-Glucosidase is a key enzyme in the release of bound aromas, as it hydrolyzes glycosidic bonds to liberate terpene-derived aromatic compounds (Testa et al., 2020; Han et al., 2023).

Non-*Saccharomyces cerevisiae* represent the primary microbial sources of *β*-glucosidase in winemaking systems. These yeasts produce glycosidases exhibiting both remarkable enzymatic stability and superior hydrolytic activity compared to *Saccharomyces cerevisiae* (Varela et al., 2016; Ruiz et al., 2019; Wei et al., 2019; Su et al., 2024). The amount and properties of enzymes produced by different yeasts vary greatly, and there are also differences between specific strains (Rodríguez et al., 2004; Zhao et al., 2021). Even among yeast strains exhibiting *β*-glucosidase activity, many may still be unable to utilize glycosidic precursors as substrates under fermentation conditions (Bisotto et al., 2015; Serra et al., 2024). Several non-*Saccharomyces cerevisiae* strains have been confirmed to have *β*-glucosidase activity, including *Candida glabrata*, *Hanseniaspora u*var*um*, *Pichia kluyveri*, *Metschnikowia chrysoperlae*, and *Lachancea thermotolerans*, which can improve the content of some volatile aroma compounds such as terpenes and benzene derivatives, thereby contributing fruity and floral aroma characteristics to wine (Karabegović et al., 2022; Boban et al., 2024a). Extracellular enzyme preparations from *Pichia fermentans* significantly enhanced the liberation of varietal aroma compounds, such as terpenols, C13-norisoprenoids, and C6 compounds (Ma et al., 2017). The *β*-glucosidase enzyme derived from *Issatchenkia terricola* significantly enhanced phenolic compound concentrations by 83% and norisoprenoid levels by 65% compared to the control (Ovalle et al., 2018).

Shandong Province, characterized by a temperate monsoon climate, experiences concurrent rainfall and high temperatures during the grape growing season. Wines produced from grapes grown under these climatic conditions often lack distinctive varietal aromatic characteristics. Studies have demonstrated that glycosidically bound terpenoids in grape berries undergo substantial accumulation starting at véraison, with their concentrations progressively increasing and consistently exceeding those of free volatile forms by 3–8-fold throughout berry development (Zhang et al., 2016; D’Onofrio et al., 2017). These findings indicate that the key limiting factor in varietal aroma expression lies in the insufficient hydrolysis of glycosylated aroma precursors. To address this enological challenge by enhancing the natural enzymatic release of glycosidically-bound aroma precursors during fermentation, this study systematically screened indigenous non-*Saccharomyces cerevisiae* strains with high *β*-glucosidase production capacity from 14 distinct vineyard sites. Subsequent pure culture fermentation will be conducted to analyze the brewing potential of each strain, including the utilization of glucose and fructose, as well as the production of ethanol, glycerol, and organic acids, along with their aromatic profiles. The present study aimed to identify non-*Saccharomyces cerevisiae* that can genuinely enhance terpene-based aromas in a brewing context and to elucidate their physicochemical and metabolic characteristics, thereby establishing a scientific basis for developing targeted co-inoculation protocols with *S. cerevisiae* to achieve precise modulation of wine terpenoid profiles.

## 2 Materials and methods

### 2.1 Screening yeast with *β*-glycosidase activity

#### 2.1.1 Yeast collection

Grape samples were collected from the Penglai region in China. Information on the wine grape varieties and corresponding vineyards for all collected samples is provided in Supplementary Table S1. The collecting time period varied from August to October in 2020, 2021, and 2022. Samples were placed in sterilized bags and transported to the laboratory under dry ice protection on the same day for further experiments. The grapes were crushed under aseptic conditions, spontaneously fermented at room temperature, and sampled on days 0, 1, 3, and 7. Each sample was diluted in gradients from 10^−1^ to 10^−7^, and 100 μL of each diluent was taken and placed in the Wallerstein Laboratory (WL) nutrient medium, which was then incubated at 28°C for 3 to 5 days. For each yeast type exhibiting different colony morphology, two strains were selected and stored on solid YPD medium. These strains were then purified by culturing a single colony on a WL plate three times at 28°C for 2 ~ 3 days. The purified single colonies were stored on inclined solid YPD medium at 4°C for further research. Stored the same one at −80°C.

#### 2.1.2 Screening and identification of yeast strains with *β*-glucosidase activity

YPD solid culture is based on high-temperature sterilization at 115°C for 20 min. When the temperature drops to approximately 60°C, the membrane-filtered geniposide and sodium glutamate solution are added, mixed, and poured into the 24-well plate under sterile conditions. Each yeast was inoculated with three replicates and cultured at 28°C, and the color development was observed and recorded.

Then, PCR was performed to obtain the 26S rDNA D1/D2 sequence to identify yeasts based on a previous study (Carla et al., 2016). PCR procedure is as listed: primer are NL1 (5′-GCATATCAATAAGCGGAGGAAAAG-3′) and NL4 (5′-GGTCCGTGTTTCAAGACG G-3′); the PCR reaction conditions are as follows: pre-degeneration at 95°C for 5 min; degeneration at 95°C for 30 s, annealing at 53°C for 20 s, extension at 72°C for 1 min, which was repeated 33 times; and a complementary extension of 72°C for 10 min; reaction system is composed of 50 μL: 2 × Phanta Max MasterMix (Vazyme Biotech Co., Ltd., Nanjing, China) 25 μL, 10 μM F-primer 1 μL, 10 μM R-primer 1 μL, template DNA 4 μL, and DD water 19 μL. The PCR products were sent to Beijing Liuhe BGI Co., Ltd., Beijing, China. for sequencing. The sequence obtained was input into the NCBI website,^1^ and BLAST was used to compare the similarities of homologous sequences in order to to preliminarily determine the species status of the strain.

#### 2.1.3 Further screening of potential strains

Fermentability and aromatic properties were measured using Durham’s fermentation tube. Durham’s fermentation tube was placed upside down in a test tube containing YPD medium, ensuring that no bubbles should exist in the tubes and that it should be completely submerged in the liquid medium. After sterilization, activated yeast was inserted into the medium using Durham’s fermentation tube and incubated at 28°C for 48 h. Gas production in Durham’s fermentation tube was observed and recorded. Yeast strains with large gas production and an elegant aroma were screened. The latter refers to a balanced and harmonious aromatic profile characterized by dominant floral/fruity notes (e.g., rose, citrus, and peach), minimal off-flavors (e.g., volatile acidity and reduction), and perceived complexity. Aroma profiles were evaluated by a panel of six trained assessors (three women and three men).

*β*-Glucosidase activity was determined according to the reference method (Swangkeaw et al., 2010). Enzyme activity was defined as the amount of enzyme required by *β*-D-glucosidase crude enzyme solution to hydrolyze p-Nitrophenyl β-D-glucopyranoside (pNPG) to produce 1 μmol of pNP within 1 min at 50°C and pH 5.0. The unit of enzyme activity is expressed as U/L.

### 2.2 Fermentations

Petit Manseng [sugars 25.6 Brix, total acid 5.6 g/L, pH 3.56, yeast assimilable nitrogen (YAN) 257.36 mg N/L] grapes were harvested in 2021 from a vineyard of China Oil and Foodstuffs Corporation (COFCO) Greatwall Winery located in the Penglai wine region of China. The grapes were destemmed and crushed; then, 200 mL of must was transferred to a 250-mL glass bottle and pasteurized at 60°C for 30 min. Monoculture fermentation with non-*Saccharomyces cerevisiae* was carried out, with each bottle inoculated with an equal number of yeast cells. The initial yeast inoculation concentration in the fermentation solution was approximately 10^7^ cells/mL, as quantified using an improved Neubauer hemocytometer with appropriate dilution factors. Each kind of yeast was inoculated in triplicate and fermented at 20°C. During the fermentation process, the amount of CO_2_ loss was measured every day until the weight loss difference between the two adjacent times was less than 0.2 g, and fermentation was finished. The physicochemical indexes of the wine and the aroma composition were measured, with commercial *S. cerevisiae* CY3079 as the control.

### 2.3 Analysis of basic oenological parameters

Basic oenological parameters, including glucose, fructose, ethanol, glycerol, and organic acids, were determined using high-performance liquid chromatography (HPLC). Chromatographic working conditions included a 300 × 7.7 mm Hi-Plex H column and a mobile phase of 0.05 mol/L of sulfuric acid solution at a flow rate of 0.6 mL/min. The column temperature is 70°C, and the differential detector has a detection temperature of 30°C. The sample size is 20 μL. Standard compounds included citric acid, tartaric acid, malic acid, succinic acid, lactic acid, and glycerin (98%, Shanghai Yuanye Bio-Technology Co., Ltd., Shanghai, China), as well as acetic acid, glucose, fructose, and ethanol (99.5%, Sinopharm Chemical Reagent Co., Ltd., Shanghai, China).

### 2.4 Analysis of volatile compounds

The volatile compounds in the wines were quantified using a headspace solid-phase microextraction coupled with gas chromatography–mass spectrometry (HS-SPME-GC–MS). GC–MS analyses were conducted using an Agilent 7890A gas chromatograph linked in line with an Agilent 5977B mass spectrometer (Agilent Technologies Inc., Santa Clara, CA, United States). The separation was achieved using a DB-WAX capillary column (30 m × 0.32 mm × 0.25 μm). An 8-mL sample containing 20 μL of 4-methyl-2-pentanol (Internal standard, 2.00 g/L) and 2 g NaCl was placed in a 20-mL headspace bottle and incubated at 45°C for 10 min. The fiber (Divinylbenzene/carboxen/polydimethylsiloxane (DVB/CAR/PDMS), 50/30 µm, Supelco, Bellefonte, PA, USA) was exposed to the headspace of the bottle for 50 min and immediately desorbed in an injector at 250°C for 10 min. The operating conditions of GC were as follows: the initial temperature was set at 40°C, increased to 45°C at 1°C/min and held for 2 min, then increased again to 180°C at 3°C/min and held for 2 min, followed by a final increase to 230°C at 5°C/min. The injector and detector were set at 250°C. The sample was injected without a shunt, and the flow rate was 0.8 mL/min. The mass spectrometry was operated in electron impact ionization mode at 70 eV, with an ion source temperature of 200°C and an interface temperature of 250°C. Detection was carried out in full-scan mode at m/z between 35 and 450. Compounds were identified by comparing their retention time with MS fragmentation patterns, which were obtained from databases NIST14 and NIST20. Volatile compounds were quantified using external standard calibration curves (Supplementary Table S2), with reference standards obtained from Aladdin Reagent Co., Ltd. (Shanghai, China). The remaining compounds were semi-quantified using the following equation:

Xi=(Ai/As)×Cs ,

where Xi is the concentration of the object to be measured, Ai is the peak area of the object to be measured, As is the peak area of the internal standard, and Cs is the internal standard concentration.

### 2.5 Sensory analysis

According to Martínez-Gil et al. (2018), combined with the national standard (Standardization Administration of China, 2006), wine sensory classification evaluation description. 12 experienced wine tasters were invited to evaluate the wine samples in terms of vision (color, clarity), aroma (intensity, floral, fruity, alcohol), taste (sweetness, acidity, body, harmony, persistence), and typicality. The results are recorded on a 10-point scale, with 0 to 10 representing a gradual increase in the enhancement of the senses. Based on the quantitative description results, the wine sensory analysis radar map was drawn.

### 2.6 Statistical analysis

SPSS 27.0 and Origin 2021 statistical packages were used for data analysis. A one-way ANOVA was used for the statistical analysis of sugars, alcohols, organic acids, and volatile compounds. Duncan’s test was used to compare the mean difference, with a probability level of 0.05. Principal component analysis (PCA) was used to examine the effects of different yeasts on wine. Only two principal components (Eigenvalues >1) were extracted according to the Kaiser criterion.

## 3 Results and discussion

### 3.1 Screening and identification

**Figure 1:**
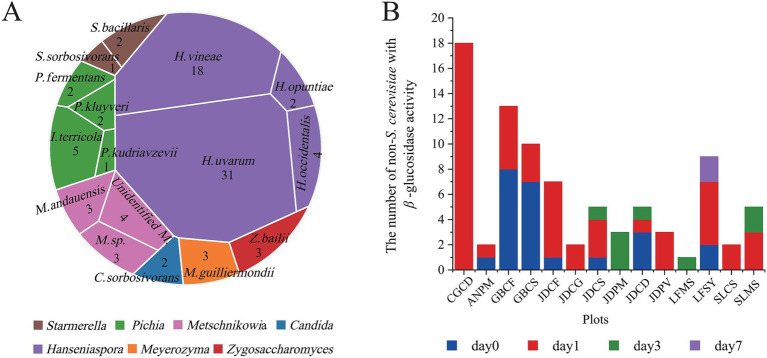
Non-*Saccharomyces cerevisiae* with *β*-glucosidase activity. **(A)** The species of non-*Saccharomyces cerevisiae* with *β*-glucosidase activity. **(B)** Changes in the number of non-*Saccharomyces cerevisiae* with *β*-glucosidase activity during spontaneous fermentation.

A total of 203 single colonies were obtained from the samples. Through the initial screening, 85 strains of non-*Saccharomyces cerevisiae* with *β*-glucosidase were obtained (Figure 1A), including 31 strains of *H. uvarum*, 18 strains of *H. vineae*, and a small number of other types, including *H. occidentalis*, *H. opuntiae*, *Starmerella bacillaris*, *I. terricola*, *Pichia kluyveri*, *Pichia kudriavzevii*, *Pichia fermentans*, *Meyerozyma guilliermondii*, *Metschnikowia andauensis*, *Zygosaccharomyces bailii*, *Starmerella sorbosivorans*, and *Candida sorbosivorans*. The yeast with *β*-glucosidase activity was mainly distributed on days 0 and 1 of spontaneous fermentation, with only a small amount present on days 3 and 7 (Figure 1B). This temporal distribution pattern may be attributed to the characteristic growth dynamics of *Hanseniaspora* species, which typically thrive during the early fermentation period (Boban et al., 2024b; Liang et al., 2023; Gurakan et al., 2022; Li S Q, et al., 2025).

### 3.2 Fermentation potential and quantification of *β*-glucosidase

After a 48-h observation of fermentation and aroma performance, 53 strains of yeast exhibiting superior fermentation performance and 26 strains of yeast with special aroma were found (Supplementary Table S3). Considering enzyme activity, fermentation, and aroma performance, one yeast of each genus was selected as the representative, and the following nine strains of non-*Saccharomyces cerevisiae* were obtained for further research (Table 1). The total *β*-glucosidase activity, quantified across the tested strains, exhibited a broad range from 37.67 U/L to 98.22 U/L. *I. terricola* CGCD-1 demonstrated the highest activity (98.22 U/L), followed by strain *Starmerella bacillaris* CGCD1-9 (87.93 U/L). Notably, *H. occidentalis* displayed significantly higher *β*-glucosidase activity (77.97 U/L) than other congeneric yeast species.

**Table 1:** Fermentation potential of nine non-*Saccharomyces cerevisiae* strains with *β*-glucosidase activity.

Strain number	Category	PGA	Fermentation capacity	Aroma	*β*-Glucosidase activity(U/L)
CGCD1-1	*H. vineae*	***	+++	Fruity and fresh	37.67
CGCD1-3	*H. uvarum*	***	+++	More fruity and fresh	39.44
CGCD1-5	*H. occidentalis*	***	++	Maltose and red dates	77.97
CGCD1-7	*H. opuntiae*	***	+++	Melons and fruits and red dates	45.71
CGCD1-4	*P. fermentans*	***	+++	Malt and beer	60.04
CGCD1-9	*S. bacillaris*	***	+++	Tea	87.93
SIVE4101^@^	*I. terricola*	***	++	Ice cream and cantaloupe	98.22
JDCD01^@^	*Z. bailii*	***	+++	Apple cider vinegar, matcha, and red bean paste	59.62
LFSY0-17	*M. andauensis*	***	+++	Preserves and licorice	49.00

Preliminary screening of *β*-glucosidase activity (PGA), measured by the color of hydrolyzed compound of geniposide: “*” low, “**” moderate, “***” high; fermentation capacity: “+” weak, “++” moderate, and “+++” intense. “@” indicates that the strain has been preserved in the China Center for Type Culture Collection (CCTCC): *Zygosaccharomyces bailii* JDCD01 (CCTCC NO: M 2022204) and *Issatchenkia terricola* SIVE4101 (CCTCC NO: M 2023794).

### 3.3 Fermentation potential of non-*Saccharomyces cerevisiae*

#### 3.3.1 Basic oenological parameters

**Figure 2:**
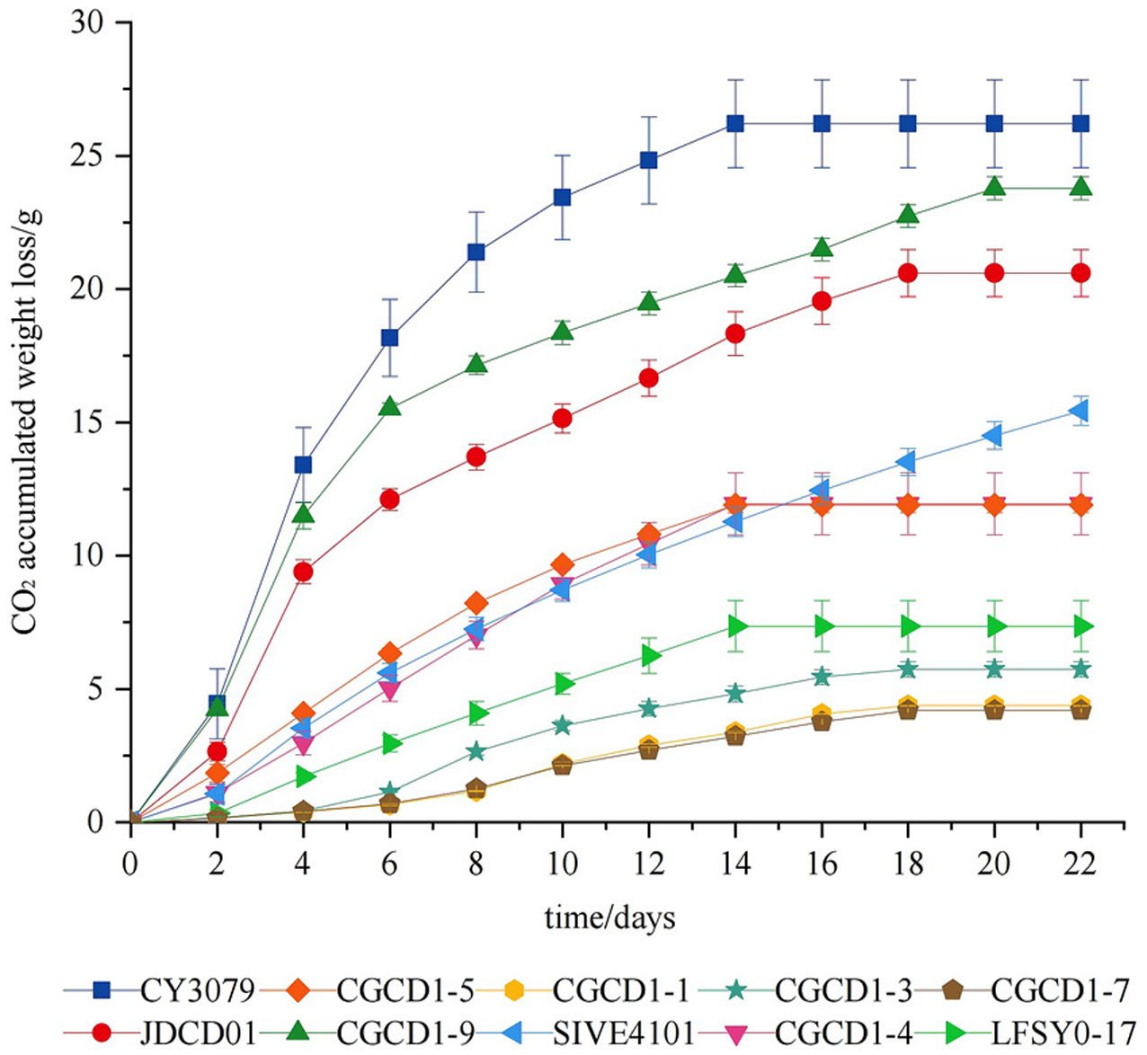
Fermentation kinetics of nine non-*Saccharomyces cerevisiae* strains compared with the control strain *S. cerevisiae* CY3079.

During alcoholic fermentation, the rate of carbon dioxide (CO_2_) release serves as a reliable indicator of both ethanol production and fermentation kinetics. Figure 2 presents the cumulative CO_2_ emission profiles of nine non-*Saccharomyces cerevisiae* strains compared with the control strain *S. cerevisiae* CY3079 throughout the fermentation process. In general, fermentation can be stopped after 20 days, except for SIVE4101, which exhibited residual CO_2_ release. Comparative fermentation kinetics revealed that all non-*Saccharomyces cerevisiae* strains exhibited significantly slower fermentation rates than the control *S. cerevisiae* CY3079. Among these strains, JDCD01 and CGCD1-9 exhibited faster fermentation rates, diverging significantly from other yeast strains after the 4th day of fermentation. Among *Hanseniaspora strains*, *H. occidentalis* CGCD1-5 exhibited significantly faster fermentation kinetics compared to the other three strains (CGCD1-1, CGCD1-3, and CGCD1-7).

The metabolic profiles of sugars, ethanol, and organic acids in non-*Saccharomyces cerevisiae* fermentations are presented in Table 2. Compared with CY3079, the total sugar consumption and ethanol production exhibited similar metabolic patterns among the nine non-*Saccharomyces cerevisiae* strains, which is consistent with the relevant reports (Błaszczyk et al., 2022; Fazio et al., 2024). Non-*Saccharomyces cerevisiae* exhibit relatively poor alcohol fermentation capacity. As the second major metabolic product of yeast, glycerol contributes to wine quality by enhancing the perception of smoothness and sweetness, while improving the complexity of aromatic and flavor profiles (Mateo and Maicas, 2016; Canonico et al., 2019; Nadai et al., 2023). JDCD01, CGCD1-4, and CGCD1-9 demonstrated enhanced glycerol biosynthesis relative to CY3079. Conversely, the *Hanseniaspora* strains’ glycerol production was significantly lower than that of CY3079. Acetic acid impairs wine quality by introducing volatile acidity faults, particularly solvent-like aromas and vinegar off-notes, when its concentration approaches the sensory threshold range of 0.7–1.1 g/L (Binati et al., 2019; Capozzi et al., 2015). In this study, all examined non-*Saccharomyces cerevisiae* strains demonstrated significantly lower (*p* < 0.05) acetic acid production compared to the *S. cerevisiae* control strain. Notably, strain LFSY0-17 displayed the most substantial reduction in acetic acid synthesis (0.13 ± 0.04 g/L), representing the lowest concentration among all tested strains. Furthermore, within the *Hanseniaspora* genus subgroup, *H. occidentalis* CGCD1-5 exhibited the minimal acetic acid yield (0.64 ± 0.07 g/L). Furthermore, strain JDCD01 showed elevated concentrations of citric acid and succinic acid relative to the control. In addition, the lactic acid produced by SIVE4101, CGCD1-9, and CGCD1-7 was higher than that of the control group. These findings underscore the metabolic diversity among non-*Saccharomyces cerevisiae* strains and their potential impact on wine quality.

**Table 2:** Content of sugar, alcohol, and organic acids in Petit Manseng wine fermented by non-*Saccharomyces cerevisiae.*

Compounds	CY3079	CGCD1-5	CGCD1-1	CGCD1-3	CGCD1-7	JDCD01	CGCD1-9	SIVE4101	CGCD1-4	LFSY0-17
Glucose	0.12 ± 0.01a	38.02 ± 0.47c	48.44 ± 1.11f	46.45 ± 0.67e	50.18 ± 0.25f	49.79 ± 1.43f	49.26 ± 0.51f	32.48 ± 0.55b	36.72 ± 0.50c	44.36 ± 1.58d
Fructose	6.26 ± 0.24b	64.48 ± 3.10e	59.69 ± 0.65d	50.99 ± 0.53c	61.52 ± 0.69d	0.32 ± 0.01a	0.11 ± 0.02a	61.96 ± 0.28d	66.41 ± 0.23e	64.19 ± 0.36e
Glycerol	10.50 ± 0.22c	6.53 ± 0.26a	6.38 ± 0.14a	8.23 ± 0.11b	6.59 ± 0.12a	11.68 ± 0.13d	15.48 ± 0.13f	10.09 ± 0.22c	14.14 ± 0.46e	6.72 ± 0.29a
Ethanol	117.34 ± 1.43 g	35.33 ± 1.39b	39.86 ± 0.28c	56.55 ± 0.95d	39.46 ± 0.73c	68.34 ± 0.87e	72.96 ± 1.20f	35.81 ± 1.66b	33.80 ± 2.62b	16.99 ± 1.19a
Citric acid	1.38 ± 0.05e	0.96 ± 0.01bc	1.02 ± 0.02 cd	1.05 ± 0.01d	1.08 ± 0.06d	0.97 ± 0.01bc	1.79 ± 0.05f	0.95 ± 0.01bc	0.90 ± 0.01b	0.81 ± 0.06a
Tartaric acid	2.06 ± 0.07a	2.14 ± 0.02ab	2.64 ± 0.07e	2.52 ± 0.07d	2.83 ± 0.03f	2.25 ± 0.04c	2.63 ± 0.04e	2.20 ± 0.04bc	2.71 ± 0.01e	2.18 ± 0.02bc
Malic acid	0.37 ± 0.02b	0.24 ± 0.07a	0.34 ± 0.02ab	0.37 ± 0.06b	0.25 ± 0.09ab	0.93 ± 0.04c	0.87 ± 0.04c	0.34 ± 0.03ab	0.32 ± 0.07ab	0.30 ± 0.04ab
Succinic acid	1.47 ± 0.01f	0.52 ± 0.05ab	0.62 ± 0.03bc	1.09 ± 0.07e	0.46 ± 0.01a	2.87 ± 0.06 g	0.89 ± 0.02d	0.63 ± 0.03bc	0.54 ± 0.01ab	0.72 ± 0.15c
Lactic acid	0.01 ± 0.00a	nd	nd	nd	0.06 ± 0.01b	nd	0.08 ± 0.01c	0.50 ± 0.01d	nd	nd
Acetic acid	0.97 ± 0.09f	0.64 ± 0.07d	0.70 ± 0.05d	0.94 ± 0.09f	0.88 ± 0.02ef	0.30 ± 0.02b	0.65 ± 0.01d	0.44 ± 0.09c	0.76 ± 0.03de	0.13 ± 0.04a

“nd” is not detected. Data with different letters (a, b, c, d, e, f, g) within each line are different according to Duncan’s multiple comparison at a level of *p* of <0.05 level.

#### 3.3.2 Wine volatile compounds

**Figure 3:**
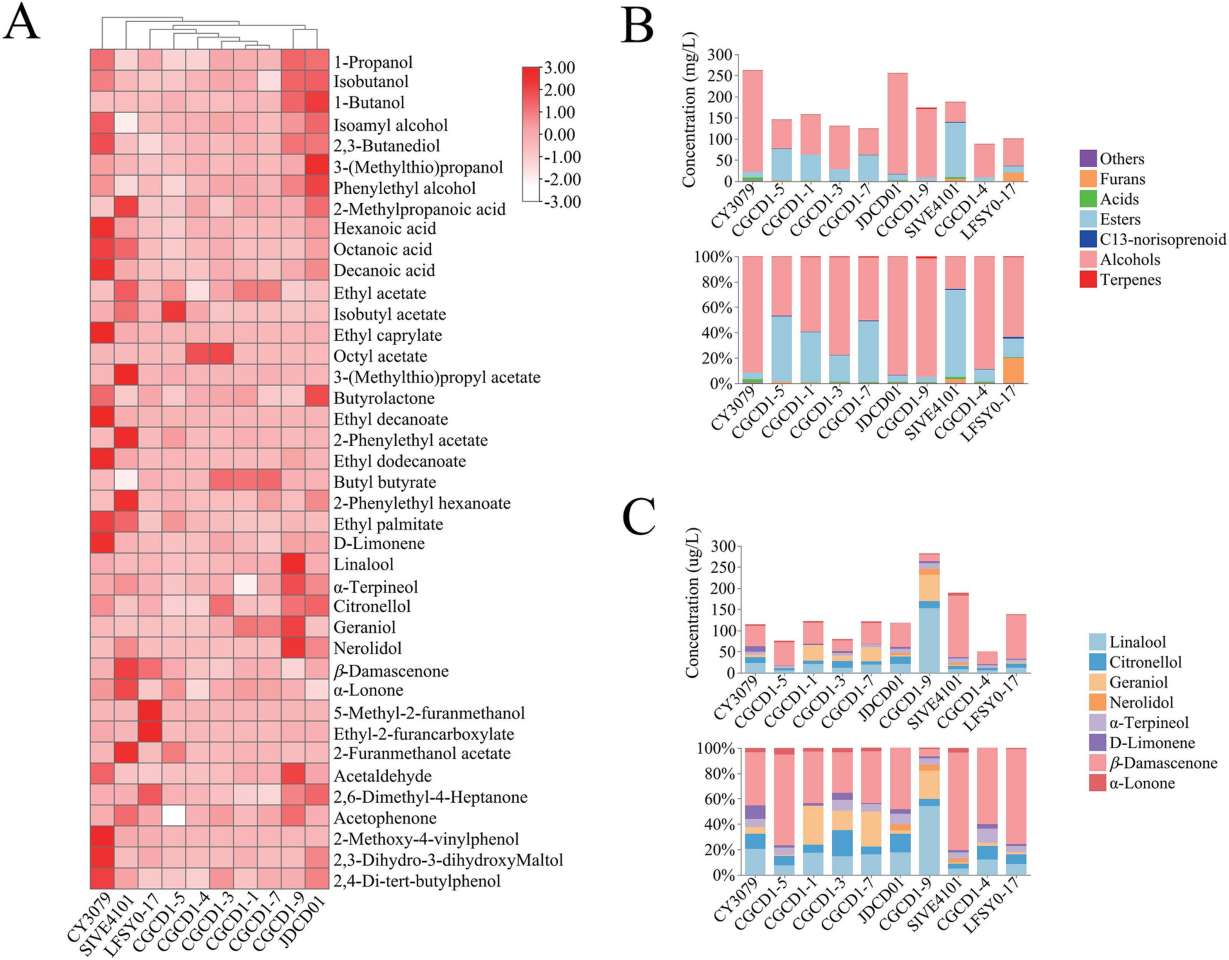
Volatile aroma compounds of non-*Saccharomyces cerevisiae.* **(A)** Heatmap of volatile aroma compounds; **(B)** the composition ratio of different types of volatile aroma components; and **(C)** the composition ratio of terpenes and C13-norisoprenoids.

A total of 40 volatile components were identified and quantified in wine samples produced by different yeast strains, including 7 alcohols, 4 acids, 12 esters, 6 terpenes, 2 C13-norisoprenoids, 3 furan derivatives, and 6 other compounds (Supplementary Table S4). To better visualize the influence of the non-*Saccharomyces* strains belonging to the diverse genera on the volatile profile of wine, the heatmap in Figure 3A shows the increased or decreased production of each volatile compound for nine non-*Saccharomyces cerevisiae* compared to the control.

Classification and statistical analysis of aroma compounds (Figure 3B) revealed that the volatile composition of most non-*Saccharomyces cerevisiae* strains exhibited similarity to CY3079, with alcohols representing the predominant fraction, followed by esters. However, LFSY0-17, CGCD1-5, and SIVE4101 demonstrated distinct compositional profiles that deviated from this pattern. Notably, LFSY0-17 showed a unique profile where furans represented the second most abundant compound class (20%) following alcohols. In contrast, CGCD1-5 demonstrated an unusual inverse ester-alcohol ratio (52% esters vs. 47% alcohols). The most striking deviation was observed in SIVE4101, where esters dominated (69%) with a substantially reduced alcohol content (26%), representing a characteristic metabolic signature distinct from that of other strains examined. In addition, compared to *S. cerevisiae* CY3079, all *Hanseniaspora* strains (CGCD1-1, CGCD1-3, CGCD1-5, and CGCD1-7) consistently exhibited enhanced ester biosynthesis capacity; CGCD1-9 showed a specific elevation in terpenoid production; SIVE4101 and LFSY0-17 promoted the accumulation of furans and C13-norisoprenoids. This study provides the first characterization of volatile aroma compounds produced by *M. andauensis* LFSY0-17. Previous research on this strain has primarily focused on its applications in biological control (Manso and Nunes, 2011; Horváth et al., 2021).

In terms of alcohols, the total alcohol content of non-*Saccharomyces cerevisiae* is lower than that of the control group, but the contents of isobutanol and phenylethyl alcohol of JDCD01 and CGCD1-9 are higher than those of the control group. This result was consistent with previous reports (Xu et al., 2017; Liu et al., 2020; Escribano-Viana et al., 2018), but slightly different from the results of Cioch-Skoneczny et al. (2021), which may be due to differences among the inter-strain. Phenylethyl alcohol has a typical rose aroma and can enrich the varietal aroma of wine (Shi et al., 2019; Li R R, et al., 2025). The phenylethyl alcohol content of JDCD01 is 40.39 mg/L, which is 2.49 times that of the control group. Given that *β*-glucosidase exhibits broad substrate specificity, some *β*-glucosidases derived from JDCD01 may catalyze the release of phenylethyl alcohol from 2-phenylethyl-*β*-D-glucopyranoside, as previously reported in the literature (Sakai et al., 2008; Han et al., 2021).

Esters are recognized as major contributors to the floral and fruity characteristics of wine and can generally be classified into three categories: fatty acid ethyl esters, acetate esters, and other esters (Katarína et al., 2017; Karabegović et al., 2022). Compared with the control group, the nine non-*Saccharomyces cerevisiae* strains tended to produce more acetate esters, likely due to the elevated activity of the ester-synthesizing enzyme, as acetate esters are formed through the condensation of acetyl-CoA with higher alcohols derived from amino acid degradation (Ehrlich pathway) or carbohydrate metabolism (Cai et al., 2014). Both four *Hanseniaspora* strains and SIVE4101 significantly enhanced ethyl acetate production. Among these, SIVE4101 exhibited the highest yield (110.56 mg/L), reaching levels 10.03-fold greater than those of the control strain CY3079 (11.02 mg/L). CGCD1-4, CGCD1-5, and SIVE4101 increased the content of isoamyl acetate, and the content of CGCD1-5 (34.59 mg/L) was 28.8 times higher than that of the control group (1.20 mg/L). Both CGCD1-4 and CGCD1-3 can increase the content of octyl acetate, with CGCD1-3 (2.79 mg/L) being higher than that of the control group (0.08 mg/L) by 34.88 times. Moreover, the content of 2-phenylethyl acetate in SIVE4101 (6.54 mg/L) is 81.75 times higher than that of CY3079 (0.08 mg/L). 3-(Methylthio) propyl acetate was not found in other yeasts, only SIVE4101 produces this substance (1.08 mg/L). All these acetate esters (except octyl acetate) exhibited odor activity values (OAVs) significantly greater than 1, indicating their substantial contributions to the floral and fruity aroma characteristics of wine.

Terpenes and C13-norisoprenoids significantly enhance the floral and sweet aroma characteristics of wines, either directly or through synergistic effects (Zhang et al., 2020; Palassarou et al., 2017; Li et al., 2024). A overall analysis revealed that CGCD1-3 and CGCD1-9 only increase the content of certain terpenes, while CGCD1-5 and LFSY0-17 solely elevate the C13-norisoprenoid levels. Notably, CGCD1-1, CGCD1-7, JDCD01, and SIVE4101 demonstrated the capacity to enhance both certain terpenes and C13-norisoprenoids, indicating substantial variation in *β*-glucosidase properties among yeast strains. Regrettably, *P. fermentans* CGCD1-4 failed to exhibit any enhancement of terpenoid or C13-norisoprenoid compounds under fermentation conditions. The potential mechanisms underlying this phenomenon may include the inhibition of enzymatic activity by elevated fermentation sugar concentrations or the intracellular localization of the enzyme, which restricts access to extracellular aroma precursors due to the impermeability of the plasma membrane to these compounds (Bisotto et al., 2015; Huang et al., 2021). Among the four *Hanseniaspora* strains (Figure 3C), CGCD1-1, CGCD1-3, and CGCD1-7 increased geraniol proportions, while *H. occidentalis* CGCD1-5 specifically enhanced *β*-damascenone percentages. Additionally, CGCD1-3 elevated citronellol and *α*-terpineol fractions. These findings align with previous reports that *β*-glycosidase from *H. uvarum* boosts terpene and C13-norisoprenoid concentrations in wine (Tristezza et al., 2016; Sun et al., 2018).

The investigated terpenoid compounds exhibited particularly low sensory thresholds, meaning even minor changes in their concentrations could significantly impact the wine’s aromatic characteristics (Han et al., 2021). Quantitative analysis revealed that linalool, citronellol, geraniol, *β*-damascenone, and *β*-ionone all demonstrated odor activity values (OAVs) exceeding 0.1, confirming their sensory relevance. CGCD1-9 produced the highest terpenoid content (264.41 μg/L), which was 4.21-fold greater than the control (62.84 μg/L). This increase was mainly attributed to high concentrations of linalool (153.31 μg/L), geraniol (62.67 μg/L), citronellol (16.22 μg/L), and α-terpineol (14.11 μg/L), which were 6.52-, 10.09-, 1.19-, and 1.98-fold higher than the control, respectively. Additionally, nerolidol (13.28 μg/L) was detected, whereas it was absent in the control. For C13-norisoprenoids, SIVE4101 achieved the highest production at 152.53 μg/L (2.96 × control), with *β*-damascenone (146.09 μg/L, 3.05×) and *β*-ionone (6.44 μg/L, 1.81×) showing marked increases. Similarly, *H. occidentalis* CGCD1-5 also enhanced both *β*-damascenone and *β*-ionone levels. *M. andauensis* LFSY0-17 produced 103.17 μg/L *β*-damascenone, indicating a statistically significant increase (*p* < 0.05). These results suggested that non-*Saccharomyces* strains could improve the concentration of terpene and *β*-damascenone, thus providing a fruity and floral flavor.

Furan aroma compounds are widely found in cigarettes, sauce-flavored liquor, and wine and play an active role in the aroma of tobacco and wine (Ivić et al., 2024; Cai et al., 2014; Jasmins et al., 2023). In addition, three strains of yeast were high-yielding for furan aroma. The content of ethyl 2-furancarboxylate in LFSY0-17 was 21.32 times that of CY3079. The content of 2-furanmethanol acetate in CGCD1-5 and SIVE4101 was 11.15 times and 41.69 times that of CY3079, respectively. Furfuryl acetate has a fruit flavor, and ethyl furoate has a barbecue flavor (Zhu et al., 2021).

#### 3.3.3 PCA analysis of aroma

**Figure 4:**
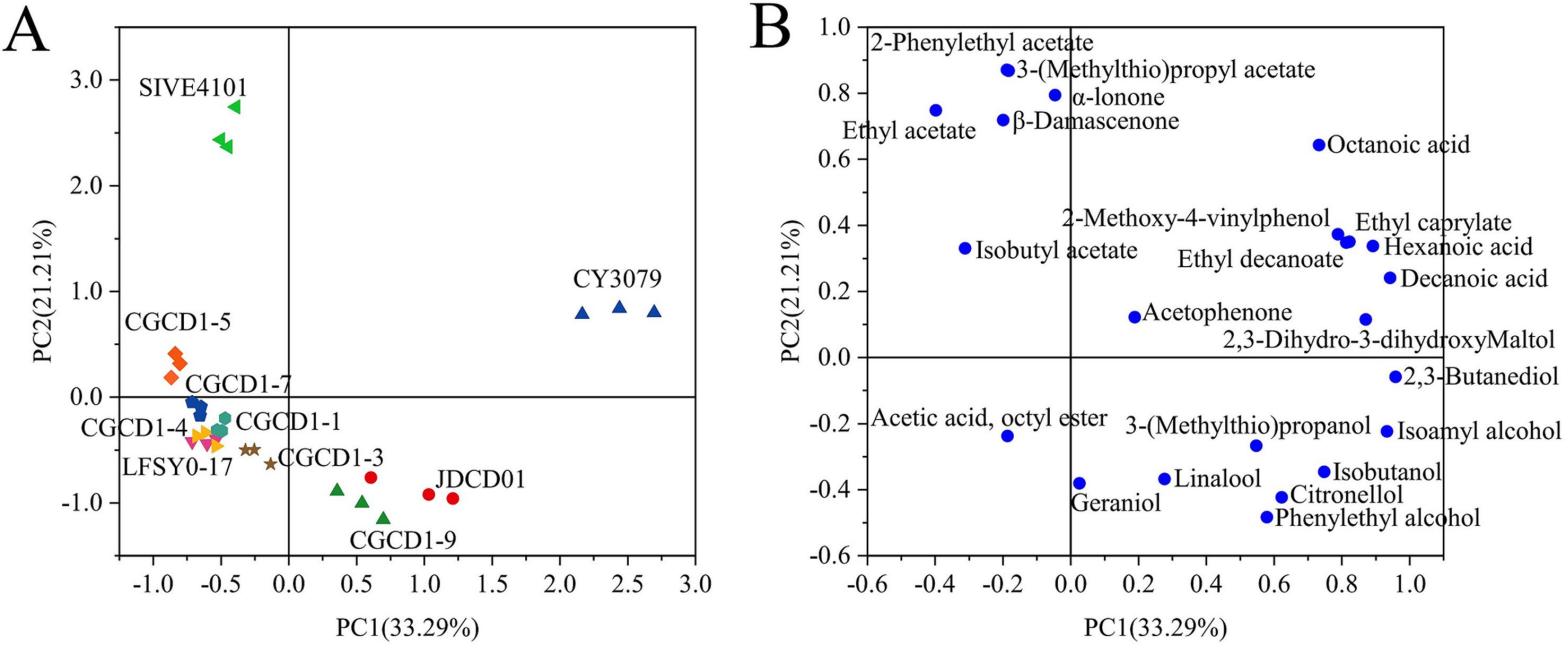
Bioplot of PCA for varietal aroma compounds from wines fermented by non-*Saccharomyces cerevisiae*. **(A)** Score plot; **(B)** loading plot.

In order to study aroma differences among strains, principal component analysis was performed on the aroma components of wine samples fermented by different yeasts (Figure 4A). The first two components explained 54.50% of the variability, with PC1 accounting for 33.29% and PC2 accounting for 21.21%. Aroma components whose content is greater than the threshold value (OAV > 0.1) represent the aroma characteristics of the wine, and the corresponding distribution is shown in Figure 4B. Principal component analysis (PCA) revealed distinct differences between Petit Manseng wines fermented with non-*Saccharomyces* yeasts and those produced with *S. cerevisiae*. The non-*Saccharomyces cerevisiae* fermentations were primarily distributed in the second, third, and fourth quadrants, whereas the *S. cerevisiae* control was localized in the first quadrant. *Non-Saccharomyces* yeasts significantly enhanced terpenes, C13-norisoprenoids, acetate esters, and higher alcohols, whereas the representative aroma compounds of *S. cerevisiae* were primarily fatty acids and ethyl esters. This trend has a certain similarity to the results of inter-group clustering in the heat map.

Among the four *Hanseniaspora* strains, CGCD1-5 was separated along PC1 and clustered in the second quadrant, whereas the other three strains, namely CGCD1-1, CGCD1-3, and CGCD1-7, were grouped in the third quadrant. SIVE4101 and CGCD1-5 were also located in the second quadrant, with ethyl acetate, 2-phenylethyl acetate, 3-(methylthio) propyl acetate, and *β*-damascenone as their key discriminant compounds. In contrast, JDCD01 and CGCD1-9 were positioned in the fourth quadrant, characterized by high levels of geraniol, linalool, and phenylethyl alcohol. Although the compounds with OAV over 0.1 (OAV > 0.1) are considered directly and individually responsible for the aroma profile of the wine, it is very important to detect the rest of the volatile compounds with lower OAVs because they contribute to the complexity of wine aroma through a synergistic effect (Karabegović et al., 2022).

#### 3.3.4 Sensory evaluation

**Figure 5:**
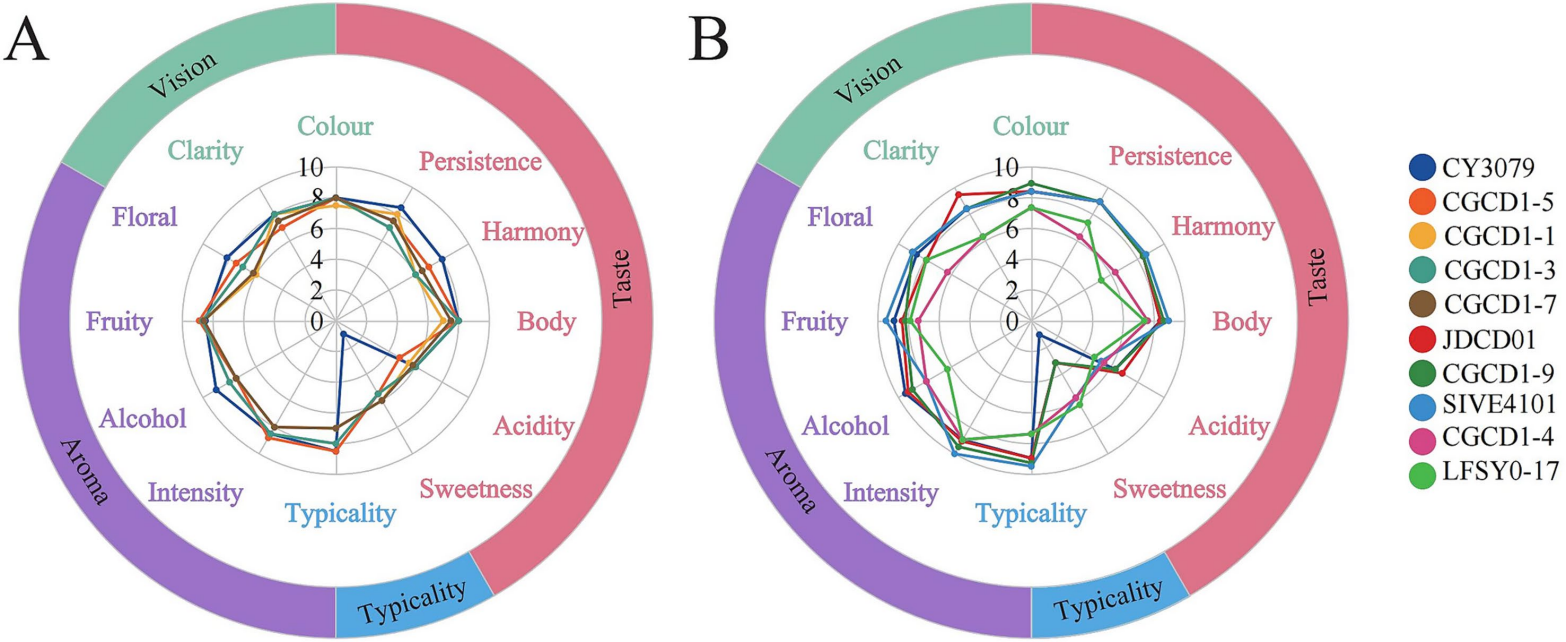
Wine sensory analysis radar map. **(A)** Sensory scores of four *Hanseniaspora* yeast strains were compared with *Saccharomyces cerevisiae* CY3079; **(B)** other strains (*P. fermentans* CGCD1-4, *S. bacillaris* CGCD1-9, *I. terricola* SIVE4101, *Z. bailii* JDCD01, and *M. andauensis* LFSY0-17) were compared with *Saccharomyces cerevisiae* CY3079.

Quantitative descriptive analysis (QDA) was conducted to evaluate the sensory characteristics of wines fermented with different yeast strains, with the results visualized in a sensory profile radar chart (Figure 4). The difference in fermentation aroma of different yeasts was demonstrated by quantitative sensory analysis. As shown in Figure 4A, wines fermented with *Hanseniaspora* exhibited higher sweetness, and the aroma was more fruity than floral. In addition, CGCD1-5 demonstrated superior performance in intensity, typicality, and body. Figure 4B reveals that the wine fermented by JDCD01 is more clarified, and CGCD1-9 has a brighter gloss in terms of appearance. From the perspective of aroma analysis, the wine fermented by SIVE4101 showed a strong floral and fruity aroma, CGCD1-9 showed more floral notes, and JDCD01 had a slightly higher intensity of aroma than the control group. From the perspective of taste analysis, due to the limited tolerance of non-*Saccharomyces cerevisiae* to alcohol, the wine fermented by it has a part of residual sugar, resulting in a sweeter taste and a lower sour taste than the control group, which may be due to the masking effect of sugar. In terms of typicality, the three strains of yeast SIVE4101, CGCD1-9, and JDCD01 had the best typicality. In addition, their total scores were all higher than those of the control group (Figure 5).

## 4 Conclusion

A total of 203 non-*Saccharomyces cerevisiae* strains were isolated from 14 plots in the Penglai wine region (China). Through primary and secondary screening, nine strains exhibiting significant *β*-glucosidase activity along with superior fermentation performance and aroma-producing capacity were selected. Fermentation trials using Petit Manseng must have demonstrated that these non-*Saccharomyces cerevisiae* strains did not adversely affect the fundamental physicochemical parameters of wine. Notably, three strains, namely CGCD1-9, CGCD1-4, and JDCD01, significantly enhanced glycerol production, which may contribute to improved wine mouthfeel and sensory quality. The selected yeast strains generated distinct volatile compound profiles, resulting in markedly different aromatic characteristics. Under oenological conditions, except *P. fermentans CGCD1-4,* eight non-*Saccharomyces cerevisiae* strains demonstrated the capacity to enhance terpenoid concentrations, potentially contributing elegant floral and fruity notes to the wine aroma profile. However, pure fermentation of non-*Saccharomyces cerevisiae* has the disadvantages of a long fermentation cycle and incomplete fermentation. To overcome these shortcomings of monoculture fermentation, co-fermentation with *S. cerevisiae* represents the optimal approach. This study characterized the oenological potential of nine indigenous non-*Saccharomyces cerevisiae* strains with high *β*-glucosidase production, providing a foundation for targeted co-inoculation strategies with *S. cerevisiae* to precisely modulate wine terpene profiles. While laboratory-scale (200 mL) fermentations provide valuable insights, industrial-scale applications may face challenges such as oxygen exposure, heat dissipation, and physical mixing. A stepwise scale-up approach (e.g., pilot-scale 100–1,000 L fermentations) should be conducted to assess the reproducibility of aroma production, fermentation efficiency, and microbial stability under conditions mimicking winery operations.

## Data Availability

The original contributions presented in the study are included in the article/Supplementary material, further inquiries can be directed to the corresponding author.

## Footnotes

^1^ https://blast.ncbi.nlm.nih.gov/Blast.cgi
