# Supplementary material for: Screening of high β-glucosidase-producing yeast strains from the Penglai wine region (China) and their fermentation performances and aroma compositions in Petit Manseng wine fermentation
Source: Front Microbiol. 2025 Aug 11;16:1653569. doi: 10.3389/fmicb.2025.1653569 (PMC12375673; doi:10.3389/fmicb.2025.1653569)
Supplement: Supplementary file 1 [file Data_Sheet_1.pdf]

Supplementary Material

Screening of High *β*-Glucosidase-Producing Yeast Strains from Penglai Wine Region (China) and Their Fermentation Performances and Aroma Compositions in Petit Manseng Wine Fermentation

Xiaohong Tang, Yan Ding, Ke Zhong, Yuxia Sun, Xiaomei Han, Zhiyu Li, Ruirui Li*

Shandong Academy of Grape/Winegrape and Wine Technological Innovation Center of Shandong Province, Jinan, Shandong, 250100, China

Email: liruirui199103@163.com

1. Supplementary Tables

Table S1 Samples of grapes collected from different vineyards

Vineyard	Grape variety	Sugars(°Brix)	Year	Abbreviation
Château Anuo	Petit Manseng	22.5	2020	ANPM
Château Guobin Penglai	Cabernet Franc	19.8	2020	GBCF
Château Guobin Penglai	Cabernet Sauvignon	21.0	2021	GBCS
Château Junding	Cabernet Franc	19.4	2020	JDCF
Château Junding	Cabernet Gernischt	19.1	2020	JDCG
Château Junding	Cabernet Sauvignon	20.8	2021	JDCS
Château Junding	Chardonnay	19.0	2020	JDCD
Château Junding	Petit Manseng	24.7	2020	JDPM
Château Junding	Petit Verdot	20.5	2020	JDPV
Domaine de Long Dai (Lafite)	Marselan	21.3	2020	LFMS
Domaine de Long Dai (Lafite)	Syrah	20.1	2020	LFSY
Château Sujialan	Cabernet Sauvignon	20.7	2021	SLCS
Château Sujialan	Marselan	21.0	2020	SLMS
COFCO Greatwall	Chardonnay	18.4	2022	CGCD

Table S2 Standard curve for volatile compounds

Compounds（mg/L）	Quantitative standard	CAS number	Standard curve	R^2	Linear range(mg/L)
Ethyl acetate	Ethyl acetate	141-78-6	y=0.0422x-0.0039	0.9997	0.19988~99.9416
Isobutanol	Isobutanol	78-83-1	y=0.0115x-0.0168	0.9996	0.12062~301.552
1-Propanol	1-Butanol	71-36-3	y=0.0234x-0.0088	0.9986	2~20
Isoamyl acetate	Isobutyl acetate	110-19-0	y=0.3977x+0.0507	0.9967	0.01~5
1-Butanol	1-Butanol	71-36-3	y=0.0234x-0.0088	0.9986	2~20
2,6-Dimethyl-4-heptanone	2,6-Dimethyl-4-heptanone	108-83-8	y=10.836x+0.1875	0.9948	0.002~0.4
Isoamyl alcohol	Isoamyl alcohol	123-51-3	y=0.0633x-0.0035	1	0.11988~299.7

Octyl acetate	Hexyl acetate	142-92-7	y=6.6674x+0.069	0.9986	0.002~1
Ethyl caprylate	Ethyl caprylate	106-32-1	y=38.541x-1.3234	0.9995	0.02009~2.5114
5-Methyl-2-Furanmethanol	Furfural	98-01-1	y=0.3152x+0.0313	0.9849	0.02~1
2,3-Butanediol	Isobutanol	78-83-1	y=0.0115x-0.0168	0.9996	0.12062~301.552
Butyrolactone	Ethyl acetate	141-78-6	y=0.0422x-0.0039	0.9997	0.19988~99.9416
Ethyl decanoate	Ethyl octanoate	106-32-1	y=38.541x-1.3234	0.9995	0.02009~2.5114
Linalool	Linalool	78-70-6	y = 17.066x + 0.0104	0.9972	0~0.04
D-Limonene	Terpinolene	586-62-9	y = 37.385x - 0.603	0.9926	0.002~0.5
α-Terpineol	α-Terpineol	98-55-5	y=35.049x-0.036	0.9979	0.0008~0.2
β-Damascenone	*β*-Damascenone	23726-91-2	y=6.0523x-0.1147	0.994	0.02~0.4
Citronellol	*β*-Citronellol	7540-51-4	y=47.161x-0.1759	0.9969	0.0008~0.2
Geraniol	Nerol	106-25-2	y = 4.1151x + 0.0158	0.9965	0~1
Nerolidol	Nerol	106-25-2	y = 4.1151x + 0.0158	0.9965	0~1
2-Phenethyl acetate	2-Phenethyl acetate	103-45-7	y=5.1868x+0.4972	0.989	0.004~5
2-Phenylethyl hexanoate	2-Phenethyl acetate	103-45-7	y=5.1868x+0.4972	0.989	0.004~5
Ethyl dodecanate	Ethyl myristate	124-06-1	y=28.449x-0.0583	0.9869	0.01~1
Butyl butyrate	Ethyl butanoate	105-54-4	y=0.5395x+0.0336	0.9972	0.004~5
α-Lonone	α-Lonone	127-41-3	y=126.17x-0.2753	0.9976	0.0008~0.4
Phenylethyl alcohol	Phenylethyl alcohol	60-12-8	y=0.2323x-0.3577	0.9989	0.11995~149.94
Ethyl palmitate	Ethyl myristate	124-06-1	y=28.449x-0.0583	0.9869	0.01~1
2-Methoxy-4-vinylphenol	2-Methoxy-4-methylphenol	93-51-6	y = 1.1714x + 0.0908	0.9926	0.05~5
3-(Methylthio)propanol	3-Methyl-1-pentanol	589-35-5	y = 0.2822x + 0.0166	0.9995	0~10

“ x ” is the concentration of the compound; “ y ” is the compound peak area/internal standard peak area.

Table S3 Fermentation capacity and aroma production capacity of yeast with *β*-glucosidase activity

Strain number	*β*-glucosidase activity	Category	fermentation capacity	Aromatic capacity
SLMS1-10	***	*H. uvarum*	+++	
SLMS1-15	**	*H. occidentalis*	+	Fruity, mineral
SLMS1-17	**	*H. occidentalis*	+	Mineral, melon, iron
SLMS2-3	***	*H. vineae*	++	
SLMS2-4	**	*H. vineae*	+	Chocolatey
SLCS1-18	***	*H. vineae*	+	
SLCS1-20	***	*H. vineae*	+	
ANPM0-1	***	*H. uvarum*	+++	
ANPM1-6	**	*H. uvarum*	+++	
GBCS0-1	***	*H. uvarum*	+++	
GBCS0-2	***	*H. uvarum*	+++	
GBCS0-3	***	*H. uvarum*	+++	
GBCS0-5	***	*H. uvarum*	+++	

GBCS0-13	**	*H. uvarum*	+++	
GBCS0-14	***	*H. uvarum*	+	Fruity, fresh
GBCS0-17	***	*H. uvarum*	+++	
GBCS1-15	***	*H. uvarum*	+++	
GBCS1-19	***	*H. uvarum*	+++	
GBCS1-20	***	*H. uvarum*	+++	
GBCF0-3	***	*H. uvarum*	+++	
GBCF0-5	***	*H. uvarum*	+++	
GBCF0-8	***	*H. uvarum*	+++	
GBCF0-10	*	*H. uvarum*	+++	
GBCF0-14	***	*H. uvarum*	+++	
GBCF0-17	**	*Unidentified M.*	+++	
GBCF0-19	**	*M. andauensis*	+++	Grain
GBCF0-20	**	*Unidentified M.*	+++	Red dates
GBCF1-7	**	*Unidentified M.*	+++	
GBCF1-10	**	*Unidentified M.*	+++	
GBCF1-11	***	*M. sp.*	+++	
GBCF1-12	***	*M. sp.*	+	
GBCF1-16	***	*M. sp.*	+	
CGCD1-1	***	*H. vineae*	+++	Fruity, fresh
CGCD1-3	***	*H. uvarum*	+++	More fruity, fresh
CGCD1-4	***	*P. Fermentans*	+++	Malt, beer
CGCD1-5	***	*H. occidentalis*	++	Maltose, red dates
CGCD1-7	***	*H. opuntiae*	+++	Melons and fruits, red dates
CGCD1-9	***	*S. bacillaris*	+++	Tea
CGCD1-10	*	*C. sorbosivorans*	+	Wheat , beer
CGCD1-13	***	*S. sorbosivorans*	+	
CGCD1-17	***	*C. sorbosivorans*	+	
SIVE4101	***	*I. terricola*	++	Ice cream, cantaloupe
CGCD1-6	***	*H. uvarum*	+	
CGCD1-11	***	*H. opuntiae*	+++	
CGCD1-15	*	*P. kluyveri*	+++	Melon, cantaloupe
CGCD1-19	*	*P. kudriavzevii*	++	
CGCD1-22	*	*I. terricola*	+	
CGCD1-23	***	*H. occidentalis*	+	Fruity, pine nuts, pear
CGCD1-26	**	*I. terricola*	+	
CGCD1-30	*	*P. kluyveri*	++	Melon, cantaloupe
CGCD1-31	**	*P. fermentans*	++	
CGCD1-32	***	*M. guilliermondii*	++	
JDCS0-4	***	*H. uvarum*	+	
JDCS1-4	***	*H. uvarum*	+++	
JDCS1-7	***	*H. uvarum*	+++	
JDCS1-18	***	*H. vineae*	+	Fruity
JDCS2-7	***	*H. uvarum*	+++	

JDCF0-21	***	*H. vineae*	+++	
JDCF1-5	***	*H. vineae*	+++	
JDCF1-6	***	*H. uvarum*	+++	
JDCF1-7	***	*H. uvarum*	+++	More fruity
JDCF1-8	***	*H. vineae*	+++	
JDCF1-9	***	*H. uvarum*	+++	
JDCF1-10	***	*H. vineae*	+++	Matcha
JDCG1-18	**	*Z. bailii*	+++	
JDCG1-22	*	*Z. bailii*	+++	
JDCD01	***	*Z. bailii*	+++	Apple cider vinegar, Matcha, red bean paste
JDCD0-6	**	*M. guilliermondii*	+	
JDCD0-7	**	*M. guilliermondii*	+	
JDPM2-15	***	*H. uvarum*	+++	
JDPM2-16	***	*H. uvarum*	+++	Roasted, caramellic
JDPM2-21	***	*H. uvarum*	+++	
JDPV1-20	*	*I. terricola*	+	
JDPV1-22	***	*H. uvarum*	+	
JDPV1-26	***	*S. bacillaris*	+++	
LFMS2-18	***	*H. vineae*	+	
LFSY0-17	***	*M. andauensis*	+++	Preserves, licorice
LFSY0-18	***	*M. andauensis*	+	
LFSY1-4	***	*H. vineae*	+++	
LFSY1-5	***	*H. vineae*	+++	
LFSY1-7	***	*H. vineae*	+++	
LFSY1-8	***	*H. vineae*	+	
LFSY1-10	***	*H. vineae*	+++	
LFSY3-13	***	*H. vineae*	+	Shrimp
LFSY3-5	***	*H. vineae*	+++	Peppermint, tea

β-Glucosidase activity: “ * ”low, “ ** ”moderate, “ *** ”high;

Fermentation capacity: “ + ”weak, “ ++ ”moderate, “ +++ ” intense.

Table S4 Volatile aroma compounds in Petit Manseng wines fermented by different yeasts

**Compounds(mg/L)**	CY3079	CGCD1-5	CGCD1-1	CGCD1-3	CGCD1-7	JDCD01	CGCD1-9	SIVE4101	CGCD1-4	LFSY0-17	Odour threshold (mg/L)	OAV	Aroma descriptor
**Total content**	262.24±17.35a	145.08±6.70cd	157.33±8.81c	129.86±5.61d	124.22±8.19de	255.14±27.28a	172.03±11.54bc	186.43±5.66b	87.92±10.57f	99.61±4.31ef			
**Alcohols**	239.15±15.97a	67.73±7.82de	93.13±7.02c	100.79±7.94c	62.43±0.90de	238.16±25.30a	162.16±10.93b	47.62±1.65e	78.04±7.72cd	63.61±8.75de			
1-Propanol #	3.82±0.34a	nd	1.07±0.29b	1.36±0.18b	0.74±0.04b	3.86±0.66a	4.44±0.33a	nd	nd	1.14±0.47b	306		Fresh, alcohol
Isobutanol #	44.50±2.85b	24.14±2.26de	32.41±1.99c	33.53±2.47c	18.97±0.08f	55.06±4.75a	53.26±4.75a	27.50±0.77cd	28.22±1.8cd	24.07±4.61de	40	＞0.1	Mild sweet, alcohol
1-Butanol #	nd	0.18±0.12c	0.15±0.11c	nd	0.07±0.1c	1.91±0.22a	1.13±0.06b	nd	nd	nd	150		Medicinal, fusel, pungency
Isoamyl alcohol#	131.10±5.98a	31.43±5.36ef	36.40±4.61de	45.52±3.72d	25.96±0.56f	111.26±8.45b	58.06±2.74c	8.36±0.48g	32.29±3.84ef	26.12±2.94f	30	＞0.1	Alcohol, harsh, bitter, banana
2,3-Butanediol #	43.39±6.83a	6.87±0.42c	10.67±0.95c	7.61±0.44c	7.48±0.25c	25.14±7.17b	25.50±3.77b	6.85±0.65c	8.57±1.02c	3.71±0.5c	120	＞0.1	Butter, creamy, chemical
3-(Methylthio)propanol # (ug/L)	157.74±0.03b	22.88±0.01de	78.12±0.02cd	37.40±0.01de	36.75±0.003de	551.04±0.08a	115.40±0.01bc	58.32±0.005cde	72.54±0.01cd	nd	382 ug/L	＞0.1	Soy sauce aroma
Phenylethyl alcohol #	16.18±0.32bc	5.09±0.89e	12.34±2.37cd	12.74±1.77cd	9.17±1.04de	40.39±4.47a	19.66±3.66b	4.45±0.09e	8.88±1.16de	8.56±1.31de	14	＞0.1	Rose, soft tommy
**Acids**	6.57±0.12a	0.03±0.01f	0.63±0.22de	1.00±0.25d	0.47±0.11e	2.25±0.23c	0.72±0.16de	3.43±0.37b	0.61±0.14de	0.34±0.02ef			
2-Methylpropanoic acid	nd	nd	0.14±0.03	nd	0.10±0.01	0.34±0.0	0.11±0.0	0.50±0.06a	0.15±0.05c	nd	8.1		Phenol, chemical, fatty

			c		c	5b	1c						
Hexanoic acid	0.92±0.02a	nd	0.09±0.04cd	0.16±0.02c	0.10±0.01cd	0.31±0.02b	0.13±0.05cd	0.28±0.09b	0.15±0.02c	0.06±0.01de	0.42	＞0.1	Cheese, rancid
Octanoic acid	3.69±0.06a	0.03±0.01g	0.24±0.13fg	0.59±0.13d	0.27±0.09fg	0.97±0.07c	0.20±0.07fg	2.33±0.23b	0.31±0.07e	0.26±0.03fg	0.5	＞0.1	Rancid, harsh, cheese, fatty acid
Decanoic acid	1.97±0.06a	nd	0.16±0.06d	0.25±0.11b	nd	0.63±0.02c	0.28±0.06c	0.32±0.02b	nd	0.02±0.01e	1	＞0.1	Sweaty
**Esters**	13.60±1.22e	75.15±1.78b	62.44±4.23c	27.06±2.92d	60.26±8.85c	13.01±1.98e	7.12±0.51e	128.77±7.06a	8.55±2.58e	15.00±4.12e			
Ethyl acetate #	11.02±0.99e	39.34±4.66c	61.14±4.22b	23.54±3.38d	53.07±2.26b	10.92±1.75e	6.76±0.50e	110.56±7.34a	3.56±1.24e	13.51±4.24e	7.5	＞0.1	Fruity, sweet
Isoamyl acetate #	1.20±0.12c	34.59±5.49a	0.29±0.13c	0.03±0.01c	0.23±0.11c	0.33±0.04c	0.11±0.02c	9.55±0.25b	2.04±0.91c	0.94±0.11c	0.03	＞0.1	Banana
Ethyl caprylate # (ug/L)	237.69±16.36a	40.38±2.40c	37.99±2.00c	36.89±1.65c	36.49±1.32c	44.95±1.18c	36.06±0.51c	57.44±0.80b	41.44±2.02c	42.46±5.89c	580 ug/L	＞0.1	Fruit, fat
Octyl acetate #	0.08±0.01b	0.03±0.01b	nd	2.79±0.49a	nd	nd	nd	nd	2.48±0.41a	nd			nf
3-(Methylthio)propyl acetate	nd	0.08±0.02b	nd	nd	0.04±0.01c	nd	nd	1.08±0.02a	nd	nd	0.007	＞0.1	Pineapple, onion, meat soup
Butyrolactone #	0.74±0.10b	nd	0.38±0.03c	0.29±0.03c	nd	1.01±0.19a	nd	nd	0.23±0.02c	0.29±0.03c	20		Creamy, creamy
Ethyl decanoate # (ug/L)	112.70±9.26a	36.83±0.95c	35.23±0.25c	35.03±0.41c	35.13±0.34c	36.54±0.15c	36.45±0.13c	42.69±0.48b	35.92±0.55c	36.33±1.35c	200 (ug/L)	＞0.1	Waxy
2-Phenylethyl acetate #	0.08±0.01c	0.83±0.08b	0.31±0.07c	0.07±0.01c	0.21±0.03c	0.21±0.02c	0.04±0.01c	6.54±0.38a	0.05±0.01c	0.04±0.01c	0.25	＞0.1	Rose, honey, tobacco

Ethyl dodecanoate # (ug/L)	9.91±0.57a	2.75±0.31c	2.49±0.25c	2.52±0.22c	2.46±0.82c	2.91±0.75c	4.18±0.25b	4.01±0.39b	2.51±0.13c	2.49±0.21c	800 (ug/L)		Sweet, floral, soapy
Butyl butyrate #	0.11±0.02b	0.12±0.01b	0.25±0.09a	0.26±0.03a	0.27±0.06a	0.13±0.02b	0.14±0.04b	nd	0.12±0.01b	0.14±0.02b			nf
2-Phenylethyl hexanoate #	nd	0.09±0.06c	nd	nd	0.22±0.05b	0.32±0.04b	nd	0.93±0.17a	nd	nd			nf
Ethyl palmitate # (ug/L)	3.92±0.25a	3.01±0.24b	2.29±0.15c	2.42±0.16c	2.35±0.06c	2.41±0.06c	2.48±0.85c	3.54±0.20a	2.47±0.06c	2.32±0.87c	2000(ug/L)		Wax, fatty
**Terpenes** (ug/L)	62.84±6.05b	17.74±2.48c	69.01±7.97b	51.51±4.13bc	68.65±6.45b	61.10±3.47b	264.41±19.06a	36.71±0.92bc	20.29±2.52c	33.69±3.26bc			
D-Limonene # (ug/L)	12.38±2.43a	1.48±0.35de	2.38±0.49cde	4.26±1.26cd	1.03±0.14e	4.20±0.42cd	4.70±1.39b	3.03±0.12cde	1.69±0.33cde	2.33±0.05cde	100(ug/L)		Lemon, citrus
Linalool # (ug/L)	23.51±3.21b	5.65±0.80e	21.37±2.02bc	11.64±0.99cde	19.68±2.44bcd	20.98±2.81bc	153.31±12.46a	9.40±0.15de	6.08±1.01e	11.94±2.85bcde	25(ug/L)	＞0.1	Muscat, flowery, fruity
α-Terpineol # (ug/L)	7.13±0.56bcd	4.12±0.85d	nd	6.78±0.71bcd	7.08±1.76bcd	9.74±0.81b	14.11±2.07a	9.18±0.88bc	5.72±0.82cd	6.39±0.53bcd	250(ug/L)		Lemon
Citronellol # (ug/L)	13.61±0.47ab	5.57±0.75d	7.74±1.53cd	16.49±1.23a	7.39±1.09cd	17.60±1.57a	16.22±2.88a	7.15±0.51cd	5.53±0.88d	10.25±1.81bc	100(ug/L)	＞0.1	Sweet, citrus-like
Geraniol # (ug/L)	6.21±0.05c	0.91±0.48c	37.53±4.03b	12.34±0.97c	33.47±4.24b	2.7±0.11c	62.67±10.04a	2.28±0.10c	1.26±0.20c	2.79±0.80c	20(ug/L)	＞0.1	Rose
Nerolidol # (ug/L)	nd	nd	nd	nd	nd	5.88±0.85b	13.28±1.94a	5.68±0.13b	nd	nd	700(ug/L)		Fruit, lily, flower, wood fragrance
**C13-Norisoprenoids** (ug/L)	51.45±2.15cd	57.94±0.92c	52.93±2.76cd	28.17±1.44e	52.25±8.67cd	57.02±6.23c	18.10±4.15e	152.53±11.68a	30.39±6.18de	104.01±19.33b			

β-Damascenone # (ug/L)	47.89±1.36cde	54.22±1.03cd	49.61±5.26cd	25.64±1.58ef	49.15±8.50cd	57.02±6.23c	15.92±3.66f	146.09±11.54a	30.39±6.18def	103.17±19.31b	0.05(ug/L)	＞0.1	Flowery, honey, sweet
α-Lonone # (ug/L)	3.56±0.81bc	3.73±0.34b	3.33±0.85bc	2.53±0.66cd	3.10±0.22bcd	nd	2.18±0.49d	6.44±0.20a	nd	0.84±0.04e	2.6(ug/L)	＞0.1	Violet, sweet fruity
**Furans**	1.02±0.12c	1.72±0.15c	0.46±0.05c	0.31±0.04c	0.35±0.03c	0.62±0.02c	0.64±0.12c	5.79±0.27b	0.3±0.08c	20.03±2.44a			
5-Methyl-2-furanmethanol #	nd	nd	nd	nd	nd	nd	nd	nd	nd	0.84±0.18a			nf
Ethyl 2-furancarboxylate	0.90±0.11b	0.27±0.08b	0.29±0.03b	0.28±0.03b	0.25±0.01b	0.56±0.01b	0.64±0.12b	0.37±0.01b	0.30±0.08b	19.19±2.26a			Died fruit，caramel
2-Furanmethanol acetate	0.13±0.02c	1.45±0.21b	0.17±0.03c	0.03±0.01c	0.10±0.02c	0.06±0.01c	nd	5.42±0.27a	nd	nd			Jam, banana
**Others**	1.78±0.17a	0.36±0.07d	0.54±0.15cd	0.62±0.30c	0.59±0.03c	0.98±0.07b	1.10±0.13b	0.62±0.06c	0.37±0.10d	0.49±0.08cd			
Acetophenone	0.06±0.02b	nd	0.04±0.01b	0.07±0.02ab	0.06±0.01ab	0.06±0.04b	0.09±0.02a	0.09±0.01a	0.05±0.02b	0.06±0.01ab	0.1	＞0.1	Acacia, sweet aroma
2-Methoxy-4-vinylphenol #	0.20±0.02a	nd	nd	nd	nd	nd	nd	0.02±0.01b	nd	0.02±0.01b	0.01	＞0.1	Spices, cloves, fried peanuts
2,3-Dihydro-3-dihydroxyMaltol	0.33±0.10a	nd	0.03±0.01c	nd	nd	0.13±0.02b	nd	nd	nd	0.06±0.01c	0.065	＞0.1	Floral
2,4-Di-tert-butylphenol	0.54±0.06a	0.10±0.01e	0.11±0.01e	0.29±0.07bc	0.20±0.01d	0.34±0.02b	0.19±0.04d	0.25±0.03cd	0.07±0.02e	0.09±0.03e			nf
Acetaldehyde	0.57±0.03a	0.18±0.06cd	0.29±0.16bc	0.20±0.05bcd	0.26±0.01bcd	0.33±0.02b	0.70±0.05a	0.17±0.02cd	0.17±0.05cd	0.14±0.02d	25		Spicy, pungent, green apple
2,6-Dimethyl-4-Heptanone #	0.09±0.01	0.08±0.01	0.06±0.03	0.07±0.05	0.06±0.01	0.12±0.0	0.11±0.0	0.08±0.01a	0.08±0.02a	0.13±0.01a	8.0		Green incense, jackfruit, mint

	abcd	abcd	cd	bcd	c	2ab	2abc	bcd	bcd

“ nd ” is not detected.

“ nf ” means not found aroma descriptor.

“ # ” represents that the aroma compound was quantified using an external standard curve.

“OAV > 0.1 ” indicates that the concentration of an aroma compound exceeds 10% of its sensory detection threshold in at least one sample, suggesting potential sensory relevance.

Data with different letters (a, b, c, d, e, f, g) within each line are different according to Duncan multiple comparison at $P<0.05$ level
